# The short-term effects of an integrated care model for the frail elderly on health, quality of life, health care use and satisfaction with care

**DOI:** 10.5334/ijic.1010

**Published:** 2014-12-05

**Authors:** Wilhelmina Mijntje Looman, Isabelle Natalina Fabbricotti, Robbert Huijsman

**Affiliations:** Erasmus University Rotterdam, Institute of Health Policy and Management, Rotterdam, The Netherlands; Erasmus University Rotterdam, Institute of Health Policy and Management, Rotterdam, The Netherlands; Erasmus University Rotterdam, Institute of Health Policy and Management, Rotterdam, The Netherlands

**Keywords:** frail elderly, integrated care, short-term effects

## Abstract

**Purpose:**

This study explores the short-term value of integrated care for the frail elderly by evaluating the effects of the Walcheren Integrated Care Model on health, quality of life, health care use and satisfaction with care after three months.

**Intervention:**

Frailty was preventively detected in elderly living at home with the Groningen Frailty Indicator. Geriatric nurse practitioners and secondary care geriatric nursing specialists were assigned as case managers and co-ordinated the care agreed upon in a multidisciplinary meeting. The general practitioner practice functions as a single entry point and supervises the co-ordination of care. The intervention encompasses task reassignment between nurses and doctors and consultations between primary, secondary and tertiary care providers. The entire process was supported by multidisciplinary protocols and web-based patient files.

**Methods:**

The design of this study was quasi-experimental. In this study, 205 frail elderly patients of three general practitioner practices that implemented the integrated care model were compared with 212 frail elderly patients of five general practitioner practices that provided usual care. The outcomes were assessed using questionnaires. Baseline measures were compared with a three-month follow-up by chi-square tests, *t*-tests and regression analysis.

**Results and conclusion:**

In the short term, the integrated care model had a significant effect on the attachment aspect of quality of life. The frail elderly patients were better able to obtain the love and friendship they desire. The use of care did not differ despite the preventive element and the need for assessments followed up with case management in the integrated care model. In the short term, there were no significant changes in health. As frailty is a progressive state, it is assumed that three months are too short to influence changes in health with integrated care models. A more longitudinal approach is required to study the value of integrated care on changes in health and the preservation of the positive effects on quality of life and health care use.

## Introduction

With the ageing of the population, the number of frail elderly people is increasing rapidly and the need to find effective care arrangements for this elderly group has gained importance [1, 2]. The frail elderly suffer from age-related problems in the physical, psychological and social domains of daily functioning [1, 3, 4]. Problems in these three domains often influence each other, which may lead to accumulating problems [3, 4]. Thus, the needs of the frail elderly are often part of a complex and dynamic process. Because the condition of the frail elderly declines gradually, timely detection is crucial as it may prevent further deterioration [5]. Research stresses the importance of detecting frailty, showing a strong relationship between frailty and quality of life [6] as well as severe problems such as disability, health care use and even death [4].

The current organisation of care is not adequate to address these complex and changing needs of the frail elderly. Current health care for the frail elderly is reactive, and frailty is often undetected by health professionals. About 30% of Dutch frail elderly people receive no domestic, personal, home or private care [7]. Health care is supply-orientated and the complex needs of the frail elderly are separately addressed by professionals focusing on their own discipline. Because the frail elderly have diverse needs in the areas of prevention, care, cure, residence and welfare [8] and professionals from these disciplines do not cooperate, care is fragmented. Fragmentation of care is further affected by a lack of continuity and co-ordination [9], leading to inefficient and ineffective care [10]. Reorganisation of care for the frail elderly is essential for creating a sustainable health care system in the future.

Integrated care is increasingly perceived as the way to reorganise care for the frail elderly. Integrated care is defined as ‘a well planned and well organised set of services and care processes, targeted at multidimensional needs/problems of an individual client, or a category of persons with similar needs/problems’ [11, p. 18]. The focus of integrated care is continuity; the set of services should be delivered seamlessly [12]. Moreover, integrated care aims to provide demand-driven care, directed at the needs of the individual client, even when these needs are multidimensional. Both continuous and demand-driven care must be achieved when care is delivered from various care disciplines or sectors [13].

Integrated care is expected to have a high level of effects [14]. It should result in more coherence in the care process, improve the quality of care and enhance clinical results, quality of life, system efficiency and consumer satisfaction [9, 15]. To explore whether these high expectations can be achieved, studies have focused on the value of integrated care and have shown mixed results. Some studies demonstrated positive effects on the functional abilities [16, 17] and well-being [17] of the frail elderly; however, other studies found no effect on functional abilities and showed an increase in the use of most types of care [18]. In a systematic review, the results suggested that there was a reduction in the use of health care as a result of integrated care [19]. Some studies found a decrease in hospitalisation and institutionalisation [16, 20, 21], whereas others found no effect of integrated care on health care utilisation [22, 23]. However, this growing body of evidence comes from studies that evaluated different integrated care models, including various components of integrated care [9, 19]. Moreover, the study periods differed considerably, and most studies focussed on the long-term effects, using a diverse range of non-valid outcome measures [19].

This study aims to address these shortcomings by exploring the short-term effects of a comprehensive integrated care model. The Walcheren Integrated Care Model has been developed and implemented in the Walcheren region, in the southwest region of the Netherlands. The project is part of The National Care for the Elderly Programme, which aims to improve Dutch elderly care through the support of projects that respond to the needs of the elderly. The project has been developed in collaboration with the elderly themselves. The Walcheren Integrated Care Model was initiated by the Walcheren General Practitioner Co-operation Care Group and developed in consultation with representatives of the elderly and health professionals in the region. The elderly indicated that the care process required greater coherence and co-ordination of care by a single portal near their homes, preferably the general practitioner. This became the point of departure for the Walcheren Integrated Care Model.

This study is relevant because of three reasons. First, the Walcheren Integrated Care Model is a comprehensive model. It includes several integrated care elements determined to be effective for the elderly: a single entry point system, case management, geriatric assessment with the EASYcare and multidisciplinary teams [2]. Furthermore, the model includes a network structure, multidisciplinary protocols, discussions and web-based patient files [13, 24, 25]. Another distinctive feature of the intervention is the focus on prevention to reduce the risk of severe problems in the physical, psychological and/or social domains for frail elderly people living independently. Thus far, only some of these elements have been combined into an integrated care model; hence, none of the models studied to date have been comprehensive.

Second, this study focuses on the short-term effects of integrated care. As previously stated, most studies investigate the long-term effects. Only three studies have evaluated the short-effects of integrated care models [17, 19, 26], but these models were not as comprehensive as the Walcheren Integrated Care Model. Furthermore, it is important to explore when integrated care models start to reach their aims to keep professionals enthusiastic and involved.

Third, the effects of the Walcheren Integrated Care Model were studied in terms of a broad range of valid health, quality of life and care outcomes. Health outcomes were assessed with the RAND, and ability to cope was measured with the KATZ. Diverse quality of life measures were used, including measures related to health (EQ-5D) and well-being (ICECAP). The care outcomes included the use of care and satisfaction with the provision of care.

## Methods

### Study design

The design of this study was quasi-experimental and included before and after measurements with a control group (for a more detailed description of the methods see also [27]). The study focused on frail elderly people living independently (living in their own homes or in some form of assisted living). The experimental group consisted of the elderly patients of eight general practitioners from three general practitioner practices located in the east of Walcheren who provided care according to the Walcheren Integrated Care Model. The control group consisted of the patients of six general practitioners from five general practitioner practices located in the northern, southern and western parts of Walcheren who provided care as usual.

### Participants

The study population consisted of the entire elderly patient population of the general practitioners in both the experimental and control groups. The inclusion criteria were being 75 years or older, not being on a waiting list for a nursing home, not being terminally ill with a life expectancy of less than six months and being frail. Frailty was assessed with the Groningen Frailty Indicator, a 15-item questionnaire that measures decreases in physical, cognitive, social and psychological functioning [28, 29]. The score ranges from 0 to 15. Elderly with a score of 4 or more were considered as being frail. General practitioners in the control group could not treat the included frail elderly patients differently as they were not given information on who participated in the study. As such, the chance of bias was minimised [30].

### Intervention

The Walcheren Integrated Care Model is a comprehensive integrated care model. The general practitioner functions as a coordinator of care and a partner in prevention. The general practitioner practice is a single entry point for the elderly, their informal caregivers and health professionals. The general practitioners detected frailty in their patient population with the Groningen Frailty Indicator. Elderly patients with a score of 4 or more were visited by their nurse practitioner, who assessed their functional, cognitive, mental and psychological functioning using EASYcare, an evidence-based instrument used to assess care needs [17]. The assessment was discussed in a multidisciplinary meeting, attended by the general practitioner, the nurse practitioner, a secondary-line geriatric nurse practitioner, a nursing home doctor and other professionals, depending on the care needed by the frail elderly. A multidisciplinary treatment plan was then formulated in consultation with the elderly person and his or her informal caregiver(s). Case management was provided by a specialised nurse practitioner or a secondary-line geriatric nursing practitioner, depending on the complexity of the elderly person's problems. Case management in this model implies responsibility for admittance to the required services, the planning and co-ordination of care delivery and periodical evaluation and monitoring of the treatment plan [31]. The evaluation took place in multidisciplinary meetings. The entire process was supported with web-based patient files and multidisciplinary protocols describing the responsibilities and activities of the involved professionals and case manager. The Walcheren Integrated Care Model requires task reassignment and delegation between nurses and doctors and between general practitioners, nursing home doctors and geriatricians. Consultations take place between primary, secondary and tertiary care providers. At the organisational level, a steering group serves as an umbrella organisation under which the Walcheren Integrated Model is developed and disseminated. The steering group, with representatives from all involved organisations, forms a Joint Governing Board that provides the necessary provider network. All patient representatives support the project, and the health insurer CZ is supporting the project financially.

Care as usual differs from the integrated model in many aspects. First, care as usual is reactive. Frail elderly patients consult with their general practitioner on their own initiative. The integrated model is proactive as frail elderly are preventively detected and assessed by their general practitioner. Second, care as usual is fragmented. In the Dutch health care system, the general practitioner has a gate keeper's role [32]. Frail elderly patients need a referral from their general practitioner to obtain care from primary, secondary or tertiary health professionals [8]. The referral letter and sporadic telephone calls are the means of communication between the general practitioner and the health professionals. In the same vein, the health professionals, to whom the elder is referred, only confer with each other bilaterally. In the integrated model, the communication is multilateral and care is co-ordinated in conference with each other in multidisciplinary meetings, with multidisciplinary protocols, case management and shared web-based files. During the study period, the general practitioners in the control group were not able to implement elements of the integrated model, because they were not supported financially by the health insurer to perform the integrated activities.

### Measures

The following health and care outcomes were studied, mostly with validated instruments.

Experienced health is assessed with an item from the RAND-36 that allows the frail elderly to evaluate their own health [33]. Mental health was measured using a five-item RAND-36 scale with items that question how often the respondent feels certain emotions, such as happiness or nervousness [33]. This scale has a Cronbach's alpha (*α*) of 0.79. Social functioning was measured with a RAND-36 item that asked whether social activities were hampered by physical health or emotional problems [33]. The Katz-15 was used to measure the ability to cope with activities of daily living, such as getting dressed, shopping and taking medication [34]. To assess quality of life, various instruments were used. First, a general measure of quality of life was used, which was based on the RAND-36 [33]. The second measure was the EQ-6D, which focuses on quality of life related to physical and mental health and includes six dimensions: mobility, self-care, daily activities, pain/discomfort, mood and cognitive functioning [35]. The third measure was the ICECAP, which was specifically developed to assess the quality of life related to well-being of the elderly. The ICECAP measures five dimensions of quality of life: attachment, security, role, enjoyment and control [36]. The instrument was based on Sen's capability approach which focuses on whether the elderly are able to function within these domains [37].

Health care use was measured with a questionnaire. Respondents were asked whether they used the following types of care: hospital care, visits to the general practitioner practice after office/open hours, day care, temporary admission to homes for the aged or nursing homes, alarm system, home care, paramedical care and psychosocial care. Satisfaction with the provision of care was examined with a self-developed questionnaire based on the consumer quality index [38]. In the consumer quality index, the following subscales are distinguished: client-oriented (*α* = 0.80), knowledge of care needs (*α* = 0.71), information (*α* = 0.77), joint decision making (*α* = 0.77), attention to social-emotional aspects (*α* = 0.72) and approach (*α* = 0.77).

The explanatory variable in the study was the introduction of the Walcheren Integrated Care Model. The control variables considered were gender, age, marital status (having a partner or not) and living arrangement (living independently at home or in residential care).

### Data collection

The data were collected by trained interviewers who visited the frail elderly at home. All frail elderly participants were interviewed face-to-face twice by the same interviewer who took a before measurement (T0) and an after measurement three months later (T1). All interviewers had a background in elderly care. All elderly completed the questionnaire on health outcomes and health care use. If a frail elderly patient received care from at least two care providers, they completed an additional questionnaire on their satisfaction with the provision of care.

### Methods of analysis

For each outcome measure, bivariate and multivariate analyses were performed. The bivariate analyses were applied to study whether the change between T0 and T1 differed significantly between the experimental and control group. For nominal variables, a chi-square test was performed to explore whether the proportion of change between T0 and T1 differed between the two groups. For the continuous variables, the difference between the T0 and T1 scores was calculated for each group after which an independent *t*-test was carried out to test whether the change over time differed between the two groups.

Multiple linear regression analysis was used to determine the effect of the Walcheren Integrated Care Model while taking the control variables into account. For the dichotomous variables for use of care, logistic regression was performed. The regression analysis consisted of three models to distinguish the effect of each group of variables on the specific outcome variable at T1. In model 1, the score at T0 of the specific outcome variable was included. For model 2, the control variables (gender, age, marital status and living arrangement) were added. In model 3, the Walcheren Integrated Care Model was incorporated. If the regression models were significant, then the effects of the separate variables were studied. The significance level used was *p* < 0.05.

## Results

In the experimental group, 892 elderly patients were approached to assess their level of frailty and to ask if they wanted to participate in this study. In the control group, 953 elderly patients were approached (Table 1). The response rate in both groups was approximately 80%, and 33% of the patients were considered frail. Ultimately, 222 frail elderly were included in the experimental group, and 224 were included in the control group. The loss to follow-up after three months was 17 frail elderly in the experimental group and 12 frail elderly in the control group. Therefore, the final study population included 205 frail elderly in the experimental group and 212 frail elderly in the control group.

The study population consisted of frail elderly with a mean age of 82 years and a mean Groningen Frailty Indicator score of 6 (Table 2). Women were overrepresented in both groups; 70% of the experimental group and 59% of the control group were women. The majority of the frail elderly did not have a partner, and most of the elderly were widows. Most of the frail elderly lived independently in their own homes (77% in the experimental group and 89% in the control group). The experimental group included significantly more women and more elderly in assisted living than the control group.

### Health and quality of life outcomes

The differences in health between the experimental and control groups were small (Table 3). In both groups, the health experience scores were low. On average, the frail elderly required help in the four domains of daily functioning. Their reported mental health, social functioning and general quality of life scores were good. The scores on the EQ-6D revealed that the study population experienced more problems with physical health (e.g., mobility and pain) than with mental health (e.g., cognitive functioning, anxiety and depression). They had the least problems with self-care.

The changes in health over three months were small. The only significant difference was observed for one dimension of the ICECAP. The frail elderly in the control group experienced a decrease in receiving the amount of love and friendship they desired, whereas this area was stable in the experimental group.

### Care usage

The types of care used most frequently were home care, an alarm system and meals on wheels (Table 4). The use of care did not change significantly over three months for either the experimental group or the control group.

The results for satisfaction with the provision of care were based on a smaller sample of frail elderly who received more than one type of care (66 frail elderly in the experimental group and 51 frail elderly in the control group). The frail elderly in both groups reported high levels of satisfaction with the provision of care (Table 5). Satisfaction did not change significantly over three months.

### Regression analysis

The results of the regression analysis showed that the Walcheren Integrated Care Model had little effect on health (Table 6), care usage (Table 7) and satisfaction with care (Table 8) in the frail elderly. The only significant effect was found for one dimension of the ICECAP. The frail elderly in the experimental group felt that they were better able to receive the love and friendship they desired than the frail elderly in the control group. No effect on care usage was found. The Walcheren Integrated Care Model did not influence the use of alarm systems, meals on wheels, home care and paramedical and psychosocial care. The main determinant for the outcomes after three months appeared to be the situation at baseline, which was significant for all outcome variables and may account for the high explained variance.

Moreover, the characteristics of the elderly affect many outcomes. Women are more negative about their health and are less mobile than men. The frail elderly in assisted living experience more pain and are less able to receive the love and friendship they desire. Having a partner has two negative effects: it leads to a decrease in social functioning and a decrease in doing things that make the elderly individual feel valued. Frail elderly with a partner were less likely to use meals on wheels than those without a partner. Age was an essential variable that had significant effects on both health and care outcomes. With age, the frail elderly showed decreases in health and social functioning, and they experienced more problems with coping, self-care, activities, cognitive functioning and control. Furthermore, there was a greater likelihood that these individuals used alarm systems, meals on wheels and home care.

## Discussion and conclusions

This study explored the short-term effects of a comprehensive integrated care intervention, the Walcheren Integrated Care Model, on the health, quality of life, health care use and satisfaction with care of frail elderly who were living independently. The main conclusion is that the Walcheren Integrated Care Model had only a small overall effect after three months. This study had two main findings. First, the model had a positive effect on attachment, a dimension of quality of life, which is the capability of the frail elderly to receive love and friendship. Second, health care use was not affected by the integrated care intervention. This result was deemed positive as it could be expected that the preventive element and the geriatric assessments followed up with case management would increase care consumption in the integrated care model. Besides these results, no other effects of the integrated care model were found. The effects were predominantly related to reported health, quality of life, care usage and satisfaction with care at the beginning of the experiment, followed by the age, marital status, sex, and living arrangements of the frail elderly.

Despite the lack of effects on most outcomes, the results of this study are relevant for several reasons. First, the positive effect on attachment shows that integrated models have the potential to influence the quality of life of the frail elderly. Affecting quality of life is important because it is a personal evaluation of both physical and psychosocial aspects of life made by the frail elderly [3]. The ability to stabilise quality of life implies that the frail elderly could live independently for a longer time period. This goal is not only the focus of national policy to reduce costs [7] but also the wish of the frail elderly themselves [12].

Second, the lack of impact on health care use is relevant for future choices in integrated care models. A possible concern may be that a proactive approach could lead to an increase in care usage. In the care as usual model, the elderly enter the health care system by visiting a general practitioner on their own initiative. In the Walcheren Integrated Care Model, all patients that were 75 years or older were proactively detected of frailty, and their needs were assessed to prevent future problems. Previous research shows that geriatric assessment could result in an increase in care usage [18, 39]. This study shows that this is not necessarily true because no increase in care usage can be observed in the short term. However, the limited changes in care usage could be a consequence of waiting lists and the care assignment routines in the Dutch health care system. Assigning care takes time because each patient has to be assessed individually by the Centre for Needs Assessment. So for some elderly patients, the length of time from geriatric assessment by the case manager to the actual receipt of care might have taken longer than three months. Because of this type of delay, the results regarding care usage may be slightly distorted.

Furthermore, the results may help health professionals to have more realistic expectations of integrated care. Currently, the expectations of integrated care and its value are very high [14]. This expectation also concerns health professionals who must stay involved to organise care according to the Walcheren Integrated Care Model on a daily basis. Expectations strongly affect performance [40] so it is crucial that professionals have realistic expectations of integrated care. This study shows that the expectations of professionals should be tempered to avoid disappointment in the short term.

Finally, this study shows that effects on health outcomes cannot be realised in the short term; however, this might not be surprising. Frailty is a gradual, progressive process of deterioration [4]. The Walcheren Integrated Model aims at an early detection of frailty. Thus, more time might be required to perceive changes in health. No effects on satisfaction with care were found, even though improvement in consumer satisfaction is an important aim of integrated care [9, 15]. A possible explanation may be that the frail elderly in the Walcheren region were already highly satisfied with care at the start of the study; hence, there is little room for improvement.

The first implication for future research is to adopt a more longitudinal approach to explore the effects of the Walcheren Integrated Care Model for frail elderly. When frail elderly are monitored for a longer period of time, actual changes in health are more likely to be observed, and the effects on the integrated care model on health-related outcomes could be studied more accurately. Second, future research could explore whether the proposed effects of integrated care emerge in a particular sequence. Frail elderly experience problems in the physical, psychological and social domains which also influence each other [3, 4]. For instance, by preserving attachment, quality of life could be improved in the future. To explore this suggestion as well as the full potential of the Walcheren Integrated Care Model, the follow-up period has been prolonged to 12 months.

## Figures and Tables

**Table 1.**
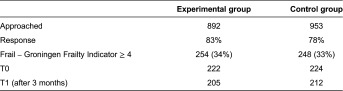
Response

**Table 2.**
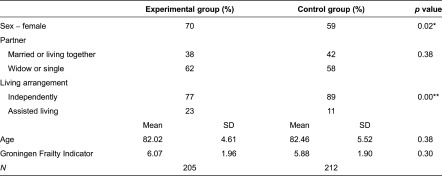
Descriptive statistics background variables

*p < 0.05; **p < 0.005.

**Table 3.**
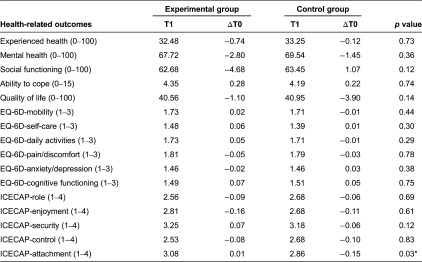
*T*-tests for health-related outcomes

*p < 0.05.

**Table 4.**
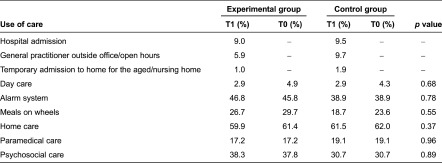
Care usage

**Table 5.**
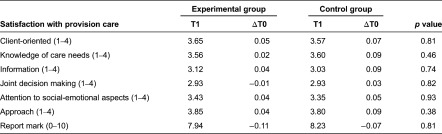
*T*-tests for satisfaction with provision of care

**Table 6.**
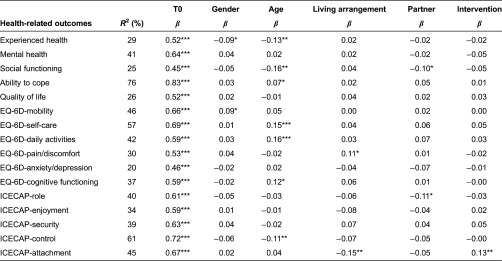
Linear regression of health-related outcomes

*p < 0.05; **p < 0.005; ***p < 0.001.

**Table 7.**
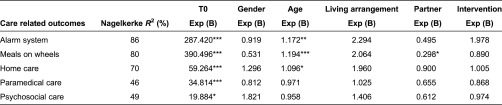
Logistic regression of care-related outcomes

*p < 0.05; **p < 0.005; ***p < 0.001.

**Table 8.**
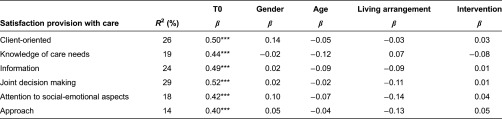
Linear regression of satisfaction with provision of care

***p < 0.001.
